# Immune Checkpoint Receptors Signaling in T Cells

**DOI:** 10.3390/ijms23073529

**Published:** 2022-03-24

**Authors:** Gianluca Baldanzi

**Affiliations:** 1 Department of Translational Medicine, University of Piemonte Orientale, 28100 Novara, Italy; gianluca.baldanzi@uniupo.it; Tel.: +39-03-2166-0527; 2 Center for Translational Research on Allergic and Autoimmune Diseases (CAAD), University of Piemonte Orientale, 28100 Novara, Italy

**Keywords:** PDL-1, SLAM, ITIM, ITSM, DGK, Src, SHP-1, SHP-2, SHIP

## Abstract

The characterization of the receptors negatively modulating lymphocyte function is rapidly advancing, driven by success in tumor immunotherapy. As a result, the number of immune checkpoint receptors characterized from a functional perspective and targeted by innovative drugs continues to expand. This review focuses on the less explored area of the signaling mechanisms of these receptors, of those expressed in T cells. Studies conducted mainly on PD-1, CTLA-4, and BTLA have evidenced that the extracellular parts of some of the receptors act as decoy receptors for activating ligands, but in all instances, the tyrosine phosphorylation of their cytoplasmatic tail drives a crucial inhibitory signal. This negative signal is mediated by a few key signal transducers, such as tyrosine phosphatase, inositol phosphatase, and diacylglycerol kinase, which allows them to counteract TCR-mediated activation. The characterization of these signaling pathways is of great interest in the development of therapies for counteracting tumor-infiltrating lymphocyte exhaustion/anergy independently from the receptors involved.

To avoid immune-mediated tissue damage and autoimmunity, the signals generated by immunoreceptors must be tightly controlled by negative signals. One of the key control mechanisms is represented by a set of receptors downmodulating the immune response in response to extracellular cues. These receptors represent immune checkpoints and are crucial for the onset of self-tolerance and chronic infection management but, at the same time, are exploited by tumors to escape the immune response [1]. Indeed, tumor-infiltrating lymphocytes often co-express multiple inhibitory receptors, which find their ligands in the tumor milieu, representing a key component for the establishment of cancer immunotolerance [2]. We will discuss the current knowledge regarding inhibitory receptor signaling in the T cell context as a base for the development of new therapies.

## 1. Introduction

In the last ten years, leveraging the immune system against tumor cells (immunotherapy) has helped pharmaceutical industries to develop immune checkpoint inhibitors. Antibodies targeting cytotoxic T-lymphocyte-associated protein 4 (CTLA-4), programmed cell death protein-1 (PD-1), and programmed cell death protein ligand-1 (PDL-1) are approved for several malignancies, and other molecules are under development. These molecules improve overall survival and reduce the risk of progression whether administered alone or in combination with other therapies [3]. Although this is a major advancement in cancer therapy, responses to immune checkpoint blockade are heterogeneous, with response rates usually below 40% and only a minority of patients achieving durable responses [4]. Intriguingly, the field is dominated by biologicals, such as monoclonal antibodies, that target surface-exposed receptors and have inherent high development and production costs, representing a challenge for health systems worldwide (as an example, see [5]). This focus on hard-to-target membrane receptors is also due to limited knowledge of the signaling pathways transducing the inhibitory signals of these checkpoint receptors. The reduced knowledge of the enzymes involved in their signaling precludes the development of small molecules capable of blocking the biological action of the whole receptor family.

**Figure 1:**
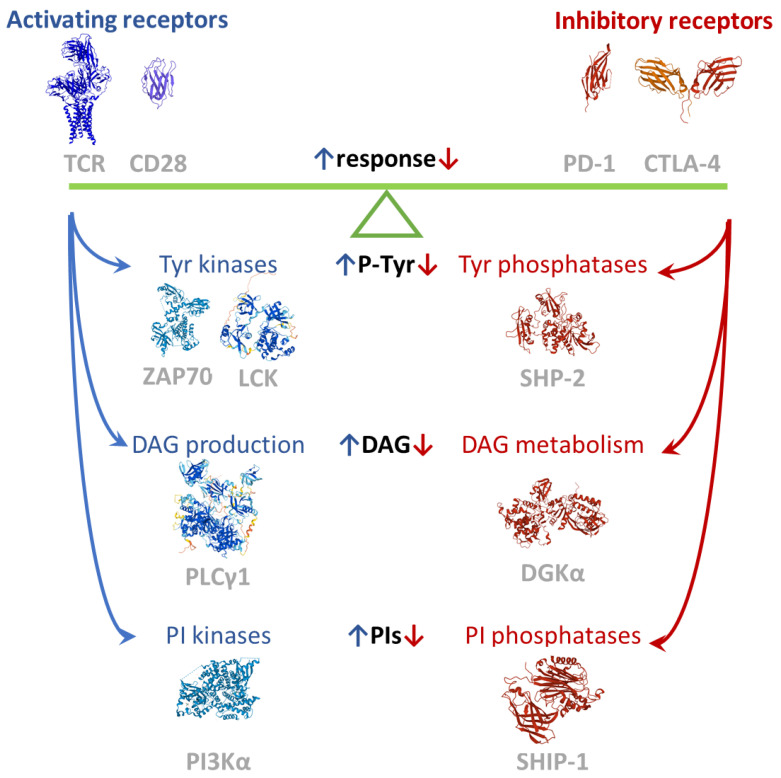
Balance of positive and negative signals tuning the immune response. Images created using RCSB PDB (www.rcsb.org accessed on 1 February 2022) and the Mol* application [10]. The TCR complex structure is 6JXR [11], the CTLA-4 partial dimeric structure is 3OSK [12], the ZAP70 structure is 4K2R [13], the SHP-2 structure is 2SHP [14], the SHIP-1 partial structure is 6XY7, the PD-1 extracellular domain is 3RRQ, and the CD28 extracellular is 6O8D (available in PDB, to be published). LCK, PLCΥ1, and DGKA models are from AlphaFold [15].

The T cell signaling machinery is dedicated to promoting the rapid expansion, differentiation, and effector response of the very small proportion of T lymphocytes which recognize non-self-antigens on antigen presenting cells (APCs). This exceptional discriminatory capability results from the intracellular integration of activating and inhibiting signals (Figure 1). Briefly, the first activating signal is delivered by the multi-subunit T cell receptor (TCR) recognizing the cognate antigen bound to the major histocompatibility complex (MHC). The activated TCR triggers the assembly of a large signalosome whose key actors are tyrosine kinases (e.g., Lck and Zap70), scaffolds (e.g., Lat, SLP76 and Themis), and phospholipase C Υ1 (PLC Υ1), but also comprises tyrosine phosphatases such as SHP1 and E3 ubiquitin ligases such as Cbl [6]. For efficient activation and anergy avoidance, a second signal must be provided by coreceptors such as CD28. CD28 reinforces TCR-driven tyrosine phosphorylation and contributes with phosphatidylinositol 3 kinase (PI3K) and Grb2 recruitment, which triggers the Akt-mTor and the Ras-MAPK pathways, respectively [7]. The best-characterized negative modulators of lymphocyte function are PD-1 and CTLA-4, which represent the paradigm of T cell inhibitory receptors. Both consistently inhibit TCR-induced cytokine secretion and proliferation and glucose uptake and metabolism [8]. Despite the presence of many other surface receptors with an inhibitory function on lymphocytes, these two are the sole receptors for which some understanding of signal transduction has been acquired. The key concept is that the cytoplasmic tails of inhibitory receptors bear the immunoreceptor tyrosine-based inhibition motif (ITIM, consensus: S/I/V/LxYxxI/V/L) and the immunoreceptor tyrosine-based switch motif (ITSM, consensus: TxYxx(V/I)). Upon engagement, both motifs are phosphorylated by Src family kinases (SFKs) and play an important role in regulating the immune system. The ITIM negatively affects signaling by recruiting phosphatases such as the SH2-domain-containing protein tyrosine phosphatases 1 and 2 (SHP-1 and SHP-2) and the SH2-containing inositol 5′-phosphatases 1 and 2 (SHIP-1 and SHIP-2) to negatively regulate cell activation. The ITSM may convey positive or negative signals by recruiting adaptors such as the signaling lymphocytic activation molecules (SLAMs)-associated protein (SAP) [9]. Less clear are the signals that link checkpoint receptors to intracellular checkpoint enzymes such as diacylglycerol kinases (DGK), a family of lipid kinases that metabolizes the key second messenger diacylglycerol (DAG), tuning down TCR signaling (Figure 1).

## 2. PD-1

**Figure 2:**
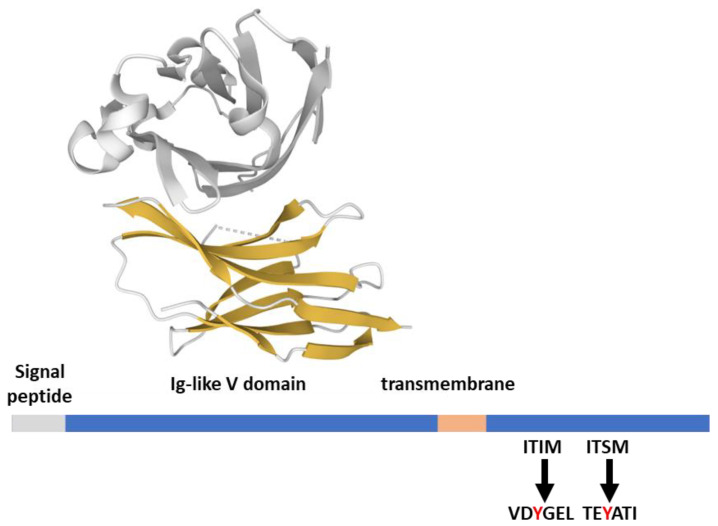
PD-1 structural features. PD-1 (288 aa) features from the Uniprot database; the extracellular domain in complex with its ligand PD-L1 (gray) is 4ZQK [37], rendered by the Mol* application [10].

The PD-1 (CD279) receptor is a member of the extended CD28 receptor family of type I transmembrane glycoproteins, but, differently from CD28, it is monomeric at the cell surface [16]. Structurally, PD-1 is composed of an extracellular immunoglobulin variable-like domain (Ig-like V), a transmembrane domain, and a cytoplasmic tail responsible for the binding of signaling and scaffolding molecules (Figure 2). The cytoplasmic tail of PD-1 contains two tyrosine motifs, an ITIM (VDY_223_GEL) and an ITSM (TEY_248_ATI; Figure 2). Both domains are phosphorylated upon PD-1 ligand engagement by the tyrosine kinase activity of Lck [17].

PD-1 is expressed on activated T and B cells, natural killer (NK) cells, monocytes, dendritic cells, and cancer cells such as melanoma. Because in lymphocytes triggering TCR and BCR induces PD-1 expression, PD-1 is generally considered to downmodulate the activity of antigen-experienced T and B cells [18]. PD-1 is activated by binding to PD-L1 (B7-H1, CD274) and with a higher affinity to PD-L2 (B7-DC, CD273). Both ligands share a conserved structure, with an extracellular portion comprising an Ig-like V and an Ig-like C domain, a transmembrane helix, and a short intracellular domain. The expression of both is interferon/cytokine-inducible but with a specific expression pattern: PD-L1 is widely expressed in hematopoietic and nonhematopoietic cells, while PD-L2 is expressed mainly on APCs [18].

A recent investigation of PD-1 effects on TCR-induced protein phosphorylation events in Jurkat cells evidenced some key features of this signaling system. The first is that in the majority of cases, PD-1 only negatively affects TCR-triggered phosphorylations on both tyrosine and serine/threonine, indicating that PD-1 acts by negatively modulating the pre-existing TCR signaling network. Functional analysis confirms a negative effect on TCR proximal signaling, resulting in a global decrease in protein production, the cell cycle, immune synapse maturation, and lymphocyte adhesion [19]. At a transcriptomic level, the effects of PD-1 are stronger on genes involved in effector function and cytokine secretion compared to proliferation and survival pathways that are more PD-1 resistant [20].

In immune cells, PD-1 signaling relies on the tyrosine phosphatase SHP-2, although some functional redundancy with SHP-1 exists, as underscored by the observation that the in vivo SHIP-2 knockdown does not affect the immune response against tumors [21,22]. The impairment of the PD-1/SHP-2 signaling axis is partially responsible for the clinical response to antibodies against PD-1 in the tumor setting [23]. However, a more transient inhibitory function of PD-1 is present in the absence of both SHP-1 and SHP-2, indicating the existence of further functional mechanisms [24]. Upon ligand binding, SHP-2 is recruited to the phosphorylated ITMS of PD-1, but the contemporary phosphorylation of the ITSM is required to induce the switch SHP-2 to the active conformation [25]. There are conflicting data on the identity of the crucial substrates of SHP-2 activity downstream of PD-1 engagement. In addition to PD-1, one of the crucial phosphatase substrates in vitro is the costimulatory receptor CD28 [17]. PD-1 accumulates at the immune synapse with PD-L2- or PD-L1-expressing APC, where it colocalize in space and time with CD28 [26]. Microscopic observation of reconstructed immune synapses reveals that in the presence of PD-L1, PD-1 and CD28 associate in microclusters surrounding the central TCR-rich zone [17], where PD-1 recruits SHP-2, promoting a decrease in CD3ζ and CD28 phosphorylation and negatively affecting TCR signaling intensity [26]. PD-1-promoted dephosphorylation of CD28 profoundly affects PI3K recruitment at the TCR signalosome, decreasing the PI3K/AKT pathway activity and its transcriptional targets, such as Bcl-xL [8]. Mutual inhibition between PD-1 and the endogenous Akt target GSK3 exists: TCR-induced phosphorylation of GSK3 decreases on PD-1 co-engagement and, conversely, GSK3 inhibition enhances T cell cytolytic functions by decreasing PD-1 expression [27]. SHP-2 is putatively responsible not only for blocking CD28 costimulatory signaling but also for the inhibition of TCR-mediated phosphorylation of ZAP70 and its association with CD3ζ, resulting in a decrease in PKCθ and ERK activation as well as in downstream IL-2 production and proliferation [28].

Several studies report but do not functionally characterize the binding of PD-1 to the inositol phosphatase SHIP-1, which putatively decreases local phosphatidylinositol 4,5-bisphosphate (PI_4,5_P2) and phosphatidylinositol 3,4,5-trisphosphate (PI_3,4,5_P3). Thus participating in the already cited decrease in the PI3K/AKT signaling axis [29].

The SAP adaptor protein is a recent addition to the PD-1 interactome. SAP is a small SH2-containing protein strongly binding to the ITSMs of SLAM family receptors. SAP inhibits PD-1 functions by high-affinity binding to its phosphotyrosines and thus competing with SHP-2 for membrane recruitment and activation. Moreover, SAP may protect the phosphotyrosines of intracellular proteins from SHP-2-mediated dephosphorylation [25]. Using SAP as bait, the same authors also demonstrated that SHP-2 dephosphorylates different targets downstream of TCR and PD-1 and added interleukin-2-inducible T cell kinase (ITK) to the list of primary targets of SHP-2 associated with PD-1 [30].

The joined inhibitory action of PD-1 on both the PI3K/AKT pathway and the MAPK pathway results in transcriptional modulation of cell cycle progression: PD-1 abrogates the expression of Skp2, a key component of the SCF–Skp2 ubiquitin ligase complex, promoting the degradation of the p27Kip1 cell cycle inhibitor. Increased levels of p27Kip1 result in a decrease in retinoblastoma protein phosphorylation and less proliferating T lymphoblasts with increased TGFβ sensitivity [31].

Another biological effect of PD-1 triggering is the inhibition of lymphocyte adhesion, which involves the SHP-2-mediated dephosphorylation of the guanine nucleotide exchange factor C3G, leading to decreased Rap-1 activation [32]. At the cellular level, PD-1 activation by its ligands counteracts the stop signal delivered by the TCR, impairing the formation of a stable immune synapse with the APC [33].

The role of PD1 in B cells is less investigated, but in this case, SHP-2 recruitment at PD-1 inhibits the B cell receptor (BCR) signaling required for the phospholipase C-γ2 (PLCγ2) activation, Ca^2+^ mobilization, and tyrosine phosphorylation of effector molecules, such as Igβ, Syk, and ERK1/2 [34].

Interestingly, the functional output of PD-1 targeting in tumors depends on the predominant PD-1 expressing subpopulation as its blockade in CD8+ effectors promote cytotoxicity while regulatory T cells promotes immunosuppression [23]. On top of that, PD-1 is also expressed in several cancer cell types, where it is induced by exposure to IFNγ. In cancer cells, it may be activated by the local PDL-1, limiting the PI3K/AKT and Ras/MAPK pathways and behaving as a tumor suppressor in an SHP-2-independent manner [35]. Some authors have also observed a positive PD-1 effect on cell proliferation and migration through SHP-2 action on the Ras pathway [36]. The presence of PD-1 in several cancer cell types and its eventual role as an oncosuppressor may contribute to the limited efficacy of therapies targeting PD-L1/PD-1 signaling.

## 3. CTLA-4

CTLA-4 interacts with the CD28 ligands B7-1 (CD80) and B7-2 (CD86) with higher affinity than with the CD28 itself [38]. The fast kinetics of these interactions and the possible formation of high-order oligomers allow for the first level of CTLA-4 inhibitory function: competition with CD28 for ligands and thus reduction of the second signal required for full T cell activation [39,40]. Moreover, CTLA-4 constitutive endocytosis my deplete APC’s B7-1 and B7-2 by transcytosis, making it less capable of T cell activation [41].

**Figure 3:**
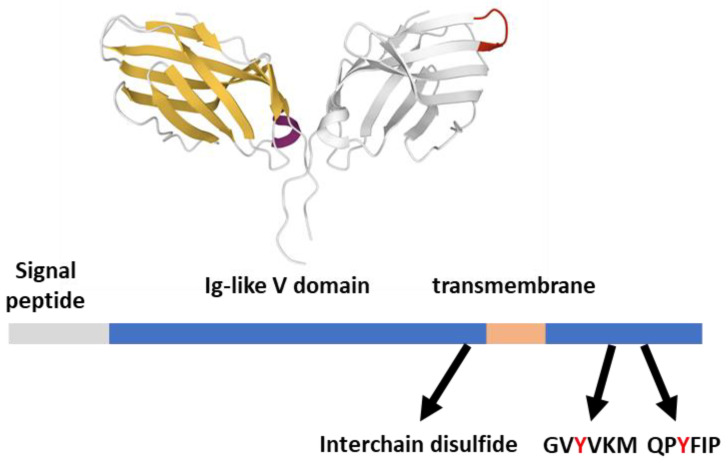
CTLA-4 structural features. CTLA-4 (233 aa) features from the Uniprot database; the Ig-like V domain unbound dimeric structure (one subunit in gray with MYPPPY ligand binding evidenced in red) is 3OSK [12], rendered by the Mol* application [10].

Structurally, CTLA-4 (CD152) features extensive similarity with CD28. Their extracellular parts feature an Ig-like V domain that allows the formation of disulfide-linked homodimers [16], while the short cytoplasmic tails can be phosphorylated by SFKs upon engagement (Figure 3). The 36-aa long CTLA-4 cytoplasmic tail is strongly conserved and contains two tyrosine substrates (Y_201_VKM and Y_218_FIP) for the kinase activity of Fyn, Lck, and possibly other kinases [42]. These two tyrosines are not canonical ITMS motifs but are involved in the CTLA-4 inhibitory function together with a proline-rich motif that could recruit SH3-domain-containing signaling molecules [43]. Despite its controversial finding in cellular models, the phosphorylation of the short CTLA-4 tail is relevant for its inhibitory action on T cell behavior in vivo, representing a second inhibitory impact of CTLA-4 [44,45].

Many effectors recruited by the CTLA-4 intracellular portion are in common with those recruited by CD28, such as PI3K and the type II serine/threonine phosphatase PP2A [46]. Conversely, the tyrosine phosphatase SHP-2 appears to be a specific interactor of the cytoplasmic domain of CTLA-4 but not of CD28, suggesting a key role of this tyrosine phosphatase in switching the signal toward an inhibitory output. Indeed, while CD28 is the prototypical second signal required for full activation, CTLA-4 co-engagement decreases early TCR signaling events, including ζ chain, Zap70, and LAT phosphorylation, as well as MAPK pathway activity, by recruiting SHP-1 or SHP-2 at the TCR signalosome [47,48]. However, CTLA-4 lacks ITMS motifs for SHP-2 binding, and direct interaction has proven hard to reconstitute in vitro after CTLA-4 phosphorylation, suggesting indirect recruitment [49].

Like PD-1, CTLA-4 engagement decreases AKT activity, but unlike in the case of PD-1, this appears to be the result of the direct action of PP2A on AKT more than the direct inhibition of PI3K activity [8]. CTLA-4 interferes with other CD28-dependent events, such as AP-1 and NF-kB activation, that drive IFNγ expression and proliferation [50]. Differently from PD-1, CTLA-4 does not impair the TCR-mediated stop signal at APC [33], and mechanistically it promotes C3G phosphorylation mediated by the SFKs member Hck as well as C3G membrane translocation, driving Rap1 activation and T cell adhesion to ICAM-1 [51]. In T cells not involved in the immune synapse, CTLA-4 may also participate in the PI3K activation required for chemokine-induced migration [52].

CTLA-4 is not present on the surface of resting T cells but increases rapidly upon stimulation through intracellular transport. Thus, it is considered a negative regulator of the first phase of T cell activation. By interacting with the clathrin adaptor complexes AP-1 and AP-2, CTLA-4 is subject to intense intracellular trafficking, and in resting cells, it is mainly stored in secretory granules. Interaction with AP-2 occurs in the surrounding of Y_201_ and favors intracellular retention and lysosomal degradation. This interaction terminates when this tyrosine is phosphorylated by kinases such as SFKs and JAK2 or the resting lymphocyte kinase [53,54], allowing CTLA-4 to move to the plasma membrane together with the binding partner Lipopolysaccharide Responsive Beige-like Anchor protein (LRBA). Thus, in the presence of its ligands, CTLA-4 translocates from intracellular stores to TCR signaling microclusters putatively located within lipid rafts [55]. At the IS, CTLA-4 concentrates in the CD3^low^ cSMAC region, where it interferes with CD28 signaling but also where transcytosis takes place [56].

CTLA-4 expression is also enhanced upon lymphocyte stimulation through the NFAT transcription factor, apart from regulatory T cells that feature a constitutive expression [57]. The constitutive presence of CTLA-4 in the T regulatory cell surface allows them to efficiently block CD28 ligands on APC and remove them by transcytosis. Note that patients with loss of function mutations in CTLA-4 or LRBA feature autoimmunity, hypogammaglobulinemia, respiratory infections, and enteropathies, in line with a homeostatic role of CTLA-4 in the control of immune function [58].

## 4. BTLA

The B and T lymphocyte attenuator (BTLA, also known as CD272) is expressed on monocytes, B cells, NK cells, and resting T lymphocytes. Its expression is upregulated in T and NK cells upon activation as well as in anergic and tumor-infiltrating T cells, while it is selectively lost by polarized Th2 cells [59].

BTLA is one of the several receptors for the TNF receptor family member herpesvirus entry mediator (HVEM, also known as TNFRSF14), which is a receptor itself, allowing bidirectional signaling. As both are expressed in T cells, heterodimerization in cis is possible and allows BTLA to repress HVEM-dependent NF-kappaB activation [60]. Conversely, HVEM binding induces BTLA phosphorylation [61], inhibiting T cell proliferation and IL-2 production [62].

**Figure 4:**
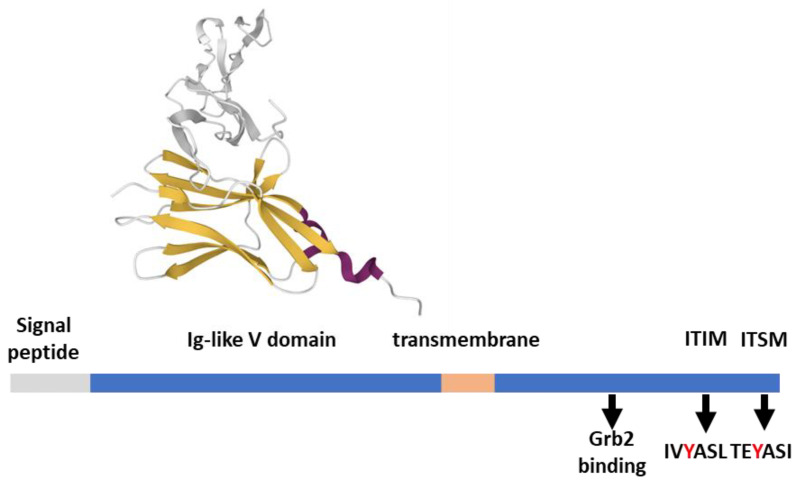
BTLA structural features. BTLA (289 aa) features from the Uniprot database; the ectodomain in complex with HVEM (gray) is 2AW2 [66], rendered by the Mol* application [10].

BTLA is related evolutionally and structurally to PD-1 and CTLA-4 (Figure 4), presenting an extracellular Ig-like V domain, a transmembrane region, and a cytoplasmic tail. Akin to PD-1, the tail of BTLA contains two additional tyrosines (Y_226_ and Y_243_) involved in Grb2 binding, an ITIM (IVY_257_ASL), and an ITSM (TEY_282_ASI)) [63,64]. These four tyrosines are required for BTLA inhibitory function, indicating that, apart from the action on HVEM, BTLA-mediated intracellular signaling is relevant. BTLA co-clusters with activated TCR at the IS and preferentially recruits SHP-1 over SHP-2 by Y_257_ and Y_282_, potently promoting dephosphorylation of both CD28 and CD3ζ [24,65]. Like what is observed for PD-1, SHP-1, and SHP-2 knockdown decreases but does not abolish the inhibition of cytokine production and proliferation by BTLA, pointing to further unidentified signal transducers [24]. Indeed, BTLA is reported to interact with several proteins, either with activating potential (such as the adaptor proteins Grb2, PI3K, Csk, and Zap70 [24,63]) or with phosphatase activity, such as the protein tyrosine phosphatase receptor type C (PTPRC) [22].

BTLA also decreases B cell proliferation and cytokine secretion by complexing with the BCR signalosome and recruiting SHP-1, resulting in a dephosphorylation of Syk kinase and a decreased activity of PLCγ2 and NF-kB [67,68].

## 5. Negative Regulators of Lymphocyte Function Outside the CD28 Superfamily

While the biological function of many other immune checkpoints is firmly established, their signal transduction is poorly understood and often depends strongly on the cellular context. In the following paragraphs, we focus on some of the immune checkpoints expressed in T cells that have been studied in some detail.

### 5.1. TIM-3

T cell immunoglobulin and mucin domain containing protein-3 (TIM-3, also known as hepatitis A virus cellular receptor 2) is expressed on Th1 CD4, CD8 (especially exhausted one), NK cells, and dendritic cells. TIM-3 presents a membrane distal Ig-like V domain involved in ligand interactions, a membrane proximal mucin domain, and a transmembrane domain connected to an intracellular cytoplasmic tail involved in phosphotyrosine-dependent signaling. TIM-3 is a promiscuous receptor suggested to bind heterologous ligands, such as phosphatidylserine, from apoptotic cells [69], carcinoembryonic antigen cell adhesion molecule 1 (CEACAM1, [70]), the alarmin protein high mobility group B1 (HMGB1), and the carbohydrate receptor galectin-9 [71]. Tim-3 is actively recruited to the IS, where it associates with other transmembrane proteins, including the CD45 and CD148 phosphatases, which may perturb IS stability [72,73].

The intracellular Tim-3 signaling in T cells depends on its cytoplasmic tail but is poorly defined and may be different between ligand-bound, plasma membrane recruited Tim-3 and the pool residing in intracellular vesicles upon exhaustion [70]. A recent proteomic study indicated a coprecipitation of Tim-3 with 37 proteins, of which 11 are dynamically regulated by pervanadate, including the E3 ubiquitin ligase CBL-B, SHP-1, and Grb2 [72]. Precedent works have added PI3Kα, Lck, interleukin inducible T cell kinase (ITK), and the adaptor HLA-B-Associated Transcript 3 (Bat3) to the list of cytoplasmatic-phosphotyrosine-dependent TIM-3 interactors [69,74,75,76]. However, the functional output of these interactions and the physiological Tim-3 agonists in T cells is debatable as it is hard to determine whether Tim-3 is resting or engaged by some ligand in the experimental system used and whether the antibodies binding to it are agonists or competing with endogenous ligands [77]. In some studies, unbound or phosphatidylserine-stimulated Tim-3 enhanced TCR signaling [69,74], while in other reports, unbound or galectin-9/CEACAM1 triggered Tim-3 suppression of TCR signaling [75,76,78]. A current working model implies a permissive Tim-3 bound to Bat3 recruiting Lck and costimulating T cells in opposition to a Tim-3 phosphorylated on tyrosines 256 and 263 by ITK switching from Bat3 to Fyn. In this model, Fyn promotes anergy through the glycosphingolipid-enriched microdomains 1 protein (PAG-1) and the inhibitory C-terminal Src kinase (CSK) [79].

### 5.2. LAIR-1

The leukocyte-associated immunoglobulin-like receptor 1 (LAIR-1, also known as CD305) is a broadly expressed inhibitory receptor for collagen and collagen domain-containing proteins, such as complement C1q. The binding of various collagen isoforms to the extracellular LAIR-1 domain inhibits the cytotoxic activity of NK cells and the activation of effector T cells. The intracellular region of LAIR-1 contains two ITIMs (Y_251_ and Y_281_) that are both phosphorylated upon LAIR-1 activation and required for SHP-1 and SHP-2 recruitment [80]. LAIR-1 Y_251_ also binds the inhibitory CSK, which is required for the negative regulation of SFKs. Indeed LAIR-1 engagement by collagen inhibits TCR-triggered phosphorylation of Lck, Lyn, and other key components in the canonical T cell signaling pathways, such as CD3ζ chain, ZAP-70, and MAPK [81].

### 5.3. LAG-3

The Lymphocyte Activation Gene-3 (LAG-3) is an inhibitory co-receptor featuring four Ig-like domains with structural similarities to CD4. Much like CD4, LAG-3 associates with the MHC class II but features a higher affinity than CD4 itself and inhibits T cell activation by interfering with the engagement of CD4 by MHC. Lag-3 is not just a CD4 competitor as it is also triggered by fibrinogen-like protein 1 (FGL1) [82], and its inhibitory function requires its intracellular region, suggesting a role in signal transduction mediated by specific sequences acting as putative interaction sites for still unknown interactors [83].

### 5.4. SLAM Family

SLAMs are a family of receptors (SLAM/CD150, CD48, Ly-9/CD229, CD84, 2B4/CD244, NTB-A/Ly108, and CRACC/CD319) expressed mainly in hematopoietic cells. These receptors signal cell proximity as they are homotypic (self-binding), except for 2B4, which recognizes CD48. SLAM family receptors present in the extracellular part an Ig-like V domain and an Ig-like C domain, followed by a transmembrane helix and a cytoplasmic tail with one or more ITMS. These ITMS may interact with either the small SH2 containing adaptors SAP and its homolog EAT-2 or inhibitory molecules, such as the protein tyrosine phosphatases SHP-1 and SHP-2, as well as the lipid phosphatase SHIP-1. On receptor engagement, SAP drives an activating signaling at those receptors by recruiting SFKs, such as Fyn and Lck [84,85], but also by competing with phosphatases such as SHP-1/SHP-2 and SHIP-1 [86,87,88]. Thus, the cells’ specific balance between these effectors determines whether SLAM receptors will deliver positive or negative signals to the receiving cells, modulating their differentiation and effector functions [89]. This double role is evidenced by the loss of function mutations of the SH2D1a gene encoding the SAP adaptor. Loss of SAP unveils the full inhibitory potential of SLAM receptors, causing X-linked lymphoproliferative disease type 1 (XLP-1, Duncan disease), a primary immunodeficiency characterized by perturbed TCR signaling and several hematological alterations, such as dysgammaglobulinemia, EBV-triggered hemophagocytic lymphohistiocytosis, and lymphomas [90]. In mature CD8 T cells, the absence of SAP decreases TCR phosphorylation and signaling strength toward Akt and MAPK pathways, impairing restimulation-induced cell death and preventing target lysis [85,91]. Similarly, CD4 T cells are deficient in IL-10 production and adhesion to B cells, required for B-cell proliferation and differentiation [89]. Finally, the absence of either SAP or SLAM receptors impairs iNKT cell development and function by decreasing TCR signaling strength [92]. In a physiological context, FOXP3 downregulates SAP expression in regulatory T cells, decreasing TCR signaling strength and rendering those cells resistant to restimulation-induced cell death [93].

## 6. Key Intracellular Transducers Downregulating TCR Signaling

From this brief description of immune checkpoint receptors, a double way of action emerges. On one hand, there are examples of competition for activating ligands (i.e., CTLA-4); on the other hand, a few identified transducers appear capable of reducing tyrosine phosphorylation and lipid signaling induced by the TCR. These enzymes are putative targets of drugs aiming to revert T cell exhaustion/anergy; this is an application explored mainly in the cancer context.

### 6.1. SHP-1 and SHP-2 in Immune Response

**Figure 5:**
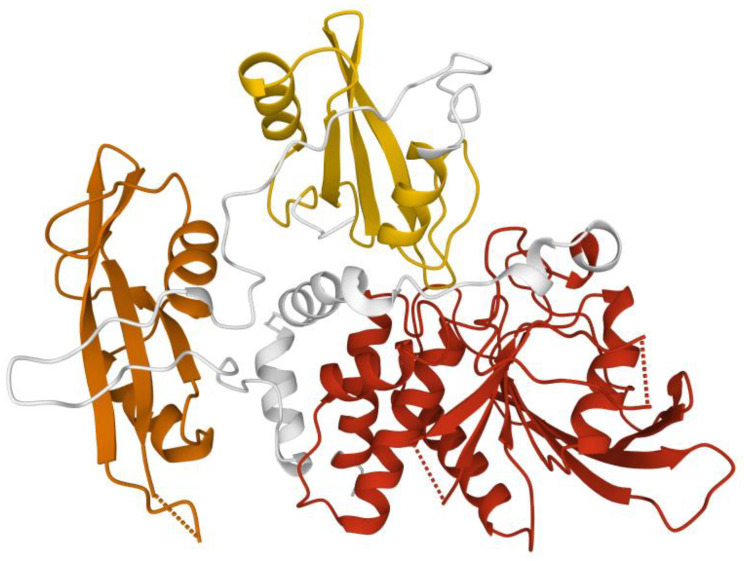
SHP-2 structure. Structure of human SHP-2 lacking the C-terminal tail (66 ammino acid). This closed conformation presents the N-terminal SH2 (yellow), C-terminal SH2 (orange), and the catalytic domain (red). Image created using RCSB PDB (www.rcsb.org accessed on 1 February 2022) and the Mol* application (8) with 2SHP [14].

Time and spatially controlled phosphotyrosine-mediated signaling arise from the strict balancing between writers (kinases) and erasers (phosphatases) influencing readers’ (SH2 and PTB domain-containing proteins) activity, as evidenced by the nearly equal number of tyrosine kinases and phosphatases in the human genome. SHP-1 and 2 are the prototypical soluble tyrosine phosphatases involved in signaling, as evidenced by the two N-terminal SH2 domains in tandem, followed by the catalytic domain and a C-terminal tail with multiple phosphorylable residues (Figure 5). These phosphatases present extensive sequence similarity and a common activation mechanism that involves tyrosine phosphorylation of the C-terminal tail and a conformational change from an inactive conformation where the N-terminal SH2 locks the catalytic domain to a relaxed conformation in which both SH2 bind to other tyrosine-phosphorylated proteins [94]. Despite this structural similarity, SHP-1 and SHP-2 consistently differ in their physiological functions and expression patterns.

SHP-1 (PTPN6) is expressed mainly in the hematopoietic system, where it acts as a negative regulator, dampening various signaling cascades [95]. In T cells, SHP-1 decreases several TCR-induced pathways, mediating cytokine production and proliferation, helping in discriminating between strong and weak agonists [96,97]. SHIP-1 also negatively modulates cytokine-induced STAT signaling, adjusting T cell differentiation and survival [98]. Indeed, motheaten mice with a mutated SHP-1 gene featured enhanced TCR signaling with severe hematopoietic disruption, chronic inflammation, and autoimmunity [99]. SHP-1 is recruited by adaptors such as Grb2 and Themis to the TCR signalosome, targeting Lck, CD3ζ chain, and ZAP70 [100]. SHP-1 is not a pure negative regulator in T cells as in some instances, TCR stimulation uses SHP-1 to dephosphorylate the adaptor CrkII, promoting adhesion, migration, and IS formation [101].

SHP-2 (PTPN11, Syp) appears to play several biological functions, as it is also widely expressed in non-hematopoietic cells and is mainly known for positively contributing to receptors signaling outside the immune system. In particular, a downregulation of the Ras/MAPK pathway and SFKs activity upon SHP-2 deletion is described in several systems [102]. This may also be due to an adaptor function of SHP-2 mediated by its tyrosine-phosphorylated C-terminal tail. Gain-of-function mutations in SHP-2 or the Ras/MAPK pathway cause Noonan syndrome, a genetic disorder with skeletal and cardiovascular defects [103]. In the TCR signaling context, SHP-2 is considered a key component of the signalosome driving the MAPK pathway [104]. Indeed, SHP-2 deficiency decreases lymphocyte activation, proliferation, and cytokine secretion [105]. However, the effective SHP-2 role is disputed as multiple activating and inhibitory receptors require this phosphatase for signal transduction [94], suggesting that target selection and biological effects depend on the signaling complexes to which SHP-2 is recruited more than on its intrinsic specificity.

Although both SHP-1 and SHP-2 bind to the PD-1 cytoplasmatic tail, SHP-2 appears to be the predominant PD-1 and CTLA-4 binder in vitro and in cells [17]. Conversely, SHP-1 associates more strongly with BTLA and is intrinsically a stronger CD3ζ phosphatase [24]. This specificity is made possible by subtle differences in SH2 specificity between the two phosphatases and the bivalent binding of both SH2 to two ITIMs/ITSMs separated by 25 amino acids, which is required for proper activation [106].

Interestingly, SHP-2 inhibitors such as SHP099 are currently in clinical trials for cancer treatment after showing antitumor effects in preclinical models. In animal models, SHP-2 inhibition reactivates the immune response against tumors in active CD8+ lymphocytes and modifies the tumor microenvironment by suppressing M2 macrophages [107].

### 6.2. SHIP-1

The SH2 containing inositol phosphatase 1 (INPP5D) is a lipid phosphatase associated with multiple receptors in hematological cells. SHIP-1 is able to dephosphorylate in vitro both membrane phosphoinositides and soluble inositol phosphates in the 5 position [108]. The protein presents an N-terminal SH2 domain to associate phosphorylated receptors that are either inhibitory with ITIM sequences or activating with ITAM, such as the TCR itself. This is followed by a PH-related domain for PI_3,4,5_P3 binding, the catalytic domain, a C2 domain for phospholipid association, and finally, a tail rich in prolines and phosphorylable residues for protein–protein interactions. In B lymphocytes, SHIP-1 is recruited in a tyrosine-dependent manner to several immunoreceptors and downmodulates the recruitment of PI_3,4,5_P3-dependent signal transducers, such as Tec kinases, Akt, and PLCγ. The PI_3,4_P2 generated by SHIP-1 may control the recruitment of distinct effector proteins, while the interaction of the C-terminal portion with adaptor molecules (such as Shc-1, Dok-1, and Grb-2) confers an adaptor function [109]. In T cells, SHIP-1 modulates T cell basal motility and plays a minor role in the control of TCR signaling and T cell maturation [110]. In addition, SHIP-1 controls cytotoxic activity and Th1 development favoring Th2 responses, putatively by influencing cytokine signaling [111]. Interestingly, short-term treatments with SHIP-1 inhibitors potentiate T cell activity and antitumor responses [112].

SHIP-2 (INPPL1) features a wider expression pattern and a similar protein organization, with the addition of a sterile α-motif domain at the carboxyl terminus for further protein–protein interactions [113]. A few studies have targeted it in T cells, indicating an extensive functional redundancy with SHP-1 as both can block the membrane recruitment of Tec tyrosine kinases [114].

### 6.3. Diacylglycerol Kinases’ Role in T Cell Anergy and Exhaustion

**Figure 6:**
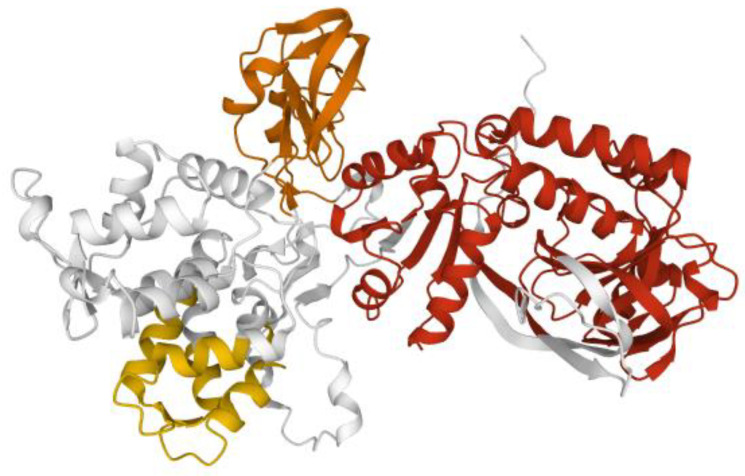
Model of DGKα. Model of DGKα structure from AlphaFold [15] rendered with the Mol* application [10] to show the two calcium binding EF hands (yellow), the two C1 domains (orange), and the split catalytic domain (red).

As shown by the common use of stimulating T cells with phorbol esters (which are nonphosphorylable DAG analogs), the PLCγ-produced DAG is one of the important second messengers induced by TCR triggering. By recruiting to the membrane RasGRP1, DAG is necessary for MAPK pathway activation [115] and, together with calcium, controls PKC activity at the IS [116]. By phosphorylating DAG, the DGK family represents a set of key intracellular negative regulators of TCR signaling and promotes T cell anergy [117,118]. In TCR signaling, two isoforms play a predominant role: DGKα (Figure 6, a calcium and tyrosine-kinase-regulated isoform also important for IL-2-induced proliferation) [119,120] and DGKζ (an isoform regulated by protein binding and phosphorylation relevant for vesicular traffic) [121]. Their role in T cell DAG metabolism is only partially similar, with DGKζ playing a quantitatively major role and DGKα s acting at IS boundaries specifically [122,123]. Not surprisingly, these enzymes are the focus of intense research aiming to restore the immunosurveillance of exhausted NK and CD8 cells against tumors [124,125,126,127,128]. DGKs knockdown is also one of the strategies to enhance CAR-T-based therapies. Even if the DGK and serotonin receptor antagonist, ritanserin, was used in clinical trials, presently, no DGK inhibitor has entered the clinic [129].

Recent studies suggest the possibility of a signaling connection between DGKα/DGKζ and immune checkpoint receptors. Indeed, both isoforms are involved in PD-1-promoted downregulation of the DAG-driven Ras/MAPK/AP1 pathway, contributing to the establishment of T cell exhaustion and PD-1 expression [130,131]. In line with this, DGKα is upregulated in tumor-infiltrating lymphocytes but also when resistance to PD-1-targeted therapies arises, and its inhibition counteracts such resistance, with the added value of directly hitting DGK-addicted cancer cells [132,133]. Interestingly, the action of the DGK family, together with PLD, produces PA, which is a signaling lipid by itself. In particular, PA is one of the lipids capable of SHP-1 binding, promoting its membrane recruitment and activation, suggesting a possible additional mechanism of T cell inhibition by DGK activity [134].

Thus, recent data indicate that DGK activity represents an intracellular immune checkpoint acting in synergy with inhibitory receptors. However, the signaling connection between the DGK family and the immune checkpoint receptors is still partially obscure and putatively isoform-specific, as every isoform has distinct regulatory domains. Strong TCR activation downmodulates DGKα expression in a PI3K/Foxo-dependent pathway [135] but also leads to a rapid decrease in its activity, which is necessary for full TCR signaling [136]. Intriguingly, SAP is necessary for this rapid DGKα inhibition [137], and it is tempting to speculate that PD-1 engagement may sequester SAP, allowing for a higher DGKα activity that contributes to TCR signal inhibition. Indeed DGKα inhibitors are capable of correcting defective cytokine synthesis and resistance to apoptosis in CD8 cells of XLP-1 patients in vitro, limiting T cell expansion in vivo [138].

## 7. Conclusions and Future Perspectives

The observation that the critical signal transducers of immune checkpoint receptors, such as SHP-1 and SHIP-1, are also central nodes of the TCR signalosome [6] suggests the idea of signaling integration as a result of fine-tuning signaling complex assembly and activity. This notion is reinforced by the observation that inhibitory receptors, such as PD-1, mainly modulate TCR signals more than generating specific ones [19]. The concept of the inhibitory receptor itself is challenged by instances such as CD5, capable of triggering both positive signaling pathways (PI3K/Akt) and negative ones (SHIP-1). The resulting effects depend on the cellular context as CD5 negatively affects T cell development while enhancing effector functions [139].

Unfortunately, we still have only a preliminary knowledge of the mechanisms governing the phosphorylation levels of key tyrosine residues or the concentrations of second messengers such as DAG, a phosphoinositide (Figure 1). Nevertheless, some cues are emerging from quantitative interactomic data even though a system biology reconstruction of the immune signaling network after physiological stimulation is missing [9,72]. Such a model, eventually supplemented by omic studies on signaling metabolites in T cells, would be precious for evaluating the output of knocking down specific components for therapeutical purposes.

Despite a limited understanding of the signaling context, the limited number of signal transducers employed by immune checkpoint receptors attract attention because of the possibility of targeting the common inhibitory mechanism instead of the specific receptor. The most advanced target is SHP-2, whose inhibitors (either allosteric or catalytic site binders) are used in several phase I trials [140], while SHIP and DGK inhibitors are still in the preclinical phase. The existence of multiple, often divergent, roles for those signal transducers, however, renders their targeting intrinsically challenging. This is evidenced by regorafenib (Stivarga), a multikinase inhibitor that is also an activator of SHP-1 and is approved for colorectal cancer therapy [141].

Less studied but equally interesting by these mechanisms are chronic infection and sepsis, where lymphocytes feature signs of exhaustion and immune response dysfunction (immune paralysis), contributing to fatal outcomes [142,143,144]. Thus, persistent viral infections and sepsis may represent a novel application area for immune checkpoint research and for the development of small molecules based targeted therapies (i.e., drug repurposing attempt in [145]).
